# Variant Transthyretin Amyloidosis (ATTRv) in Hungary: First Data on Epidemiology and Clinical Features

**DOI:** 10.3390/genes12081152

**Published:** 2021-07-28

**Authors:** Zoltán Pozsonyi, Gergely Peskó, Hedvig Takács, Dorottya Csuka, Viktória Nagy, Ágnes Szilágyi, Lidia Hategan, Balázs Muk, Beáta Csányi, Noémi Nyolczas, Lívia Dézsi, Judit Mária Molnár, Anita Csillik, Katalin Révész, Béla Iványi, Fruzsina Szabó, Krisztián Birtalan, Tamás Masszi, Zsuzsanna Arányi, Róbert Sepp

**Affiliations:** 1 Department of Internal Medicine and Haematology, Semmelweis University, H-1088 Budapest, Hungary; pozsonyizoltanimre@gmail.com (Z.P.); csuka.dorottya@med.semmelweis-univ.hu (D.C.); szilagyi.agnes@semmelweis-univ.hu (Á.S.); revesz.katalin@med.semmelweis-univ.hu (K.R.); masszi.tamas@med.semmelweis-univ.hu (T.M.); 2 Division of Non-Invasive Cardiology, Department of Internal Medicine, University of Szeged, H-6725 Szeged, Hungary; takacs.hedvig88@gmail.com (H.T.); viktoriadrnagy@gmail.com (V.N.); lidiahategan@yahoo.com (L.H.); csabea88@gmail.com (B.C.); birtalan.krisztian@uzsoki.hu (K.B.); sepprobert@gmail.com (R.S.); 3 Military Hospital—State Health Centre, H-1134 Budapest, Hungary; balazsmukmd@gmail.com; 4 Gottsegen National Cardiovascular Center, H-1096 Budapest, Hungary; nyolczasnoemi@gmail.com; 5 Department of Neurology, University of Szeged, H-6725 Szeged, Hungary; dezsi.livia@med.u-szeged.hu; 6 Institute of Genomic Medicine and Rare Disorders, Semmelweis University, H-1088 Budapest, Hungary; molnar.mariajudit@med.semmelweis-univ.hu (J.M.M.); szabo.fruzsina@med.semmelweis-univ.hu (F.S.); 7 Department of Neurology, Semmelweis University, H-1088 Budapest, Hungary; anita.csillik@med.semmelweis-univ.hu (A.C.); aranyi.zsuzsanna@med.semmelweis-univ.hu (Z.A.); 8 Department of Pathology, University of Szeged, H-6725 Szeged, Hungary; ivanyi.bela@med.u-szeged.hu

**Keywords:** variant, transthyretin, amyloidosis, heart failure, polyneuropathy, ATTRHis88Arg

## Abstract

Background: Variant transthyretin amyloidosis (ATTRv) is an autosomal dominant inherited disease, where the mutation of the transthyretin gene (TTR) results in the deposition of pathogenic protein fibrils in various tissues. The mutation type influences the clinical course. Until now, no data were available on the genotype, phenotype, and prevalence of Hungarian ATTRv patients. The aim of our study was to assess the prevalence, regional distribution, genotypes, and phenotypes of Hungarian patients with ATTRv. Methods: With the collaboration of Hungarian regional and university centers, we identified patients diagnosed with ATTRv. We also searched prior publications for case studies of Hungarian ATTRv patients. Results: 40 individuals in 23 families with ATTRv were identified within the borders of Hungary. At the time of the diagnosis, 24 of them were symptomatic. The two most common mutations were ATTRHis88Arg (nine families) and ATTRIle107Val (8 families). ATTRVal30Met was demonstrated in 2 families, and ATTRVal122del, ATTRPhe33Leu, ATTRIle84Ser, and ATTRAsp18Gly in one family each. The median age of the symptomatic patients at the time of clinical diagnosis was 65 years. The most common clinically significant organ involvement was restrictive cardiomyopathy, found in 24 patients. Polyneuropathy was diagnosed in 20 patients. A total of 19 patients showed a mixed phenotype. The leading symptom was heart failure in 8 cases (3 of them had only cardiac symptoms), polyneuropathy in 11 cases (all of them also had cardiac symptoms), and equally severe cardiac and neuropathy symptoms were present in 3 cases. Out of 24 symptomatic patients, 10 received targeted pharmacological therapy. The follow-up period ranged from 1 to 195 months. At the time of the retrospective analysis, 12 patients had already died, and 1 patient underwent heart transplantation. Conclusions: As TTR genotype influences the phenotype and clinical course of ATTRv, it is important to know the regional data. In Hungary, ATTRHis88Arg and ATTRIle107Val are the most common mutations in ATTRv, both presenting with mixed phenotype, but the median age at the time of the diagnosis is 9 years lower in patients with ATTRHis88Arg than in patients with ATTRIle107Val.

## 1. Introduction

In systemic amyloidosis extracellular deposition of amyloid fibrils in certain tissues formed by different proteins leads to distinct clinical manifestations. More than 30 proteins are able to form amyloid in the human body and damage the affected tissues. The three most common forms are amyloid light chain (AL) amyloidosis associated with plasma cell dyscrasias, amyloid A (AA) amyloidosis in chronic inflammatory disorders, and transthyretin (*TTR*) amyloidosis [1]. Transthyretin can cause disease without mutation (wild-type—ATTRwt) or with certain mutations (variant—ATTRv) in the *TTR* gene. The two clinically important manifestations of ATTRwt are heart failure (HF) and carpal tunnel syndrome (CTS). ATTRwt is also called senile ATTR, due to its late presentation, with patients usually becoming symptomatic above the age of 70 or 80 years. Systemic amyloidosis is considered to be a rare disease, although ATTRwt is found in 25% of adults older than 85 years on autopsy [2]. ATTRv, which is also called hereditary ATTR due to its autosomal dominant inheritance, causes symptoms earlier than ATTRwt. More than 120 pathogenic mutations of the *TTR* gene have been identified [3]. Following the latest recommendations of the International Society of Amyloidosis (ISA) Nomenclature Committee, we use the term ATTRv [4]. Formerly, the disease was widely called familial amyloid neuropathy (ATTR-FAP), primarily in those forms, where the polyneuropathy was the leading clinical manifestation.

ATTRv mainly affects the heart and the peripheral nervous system but may involve other organs as well, such as the intestines, the eyes, or the central nervous system. CTS is also a common manifestation. The organ involvement (clinical phenotype) and the clinical course of the disease is largely dependent on the type of mutation. Some mutations primarily cause progressive polyneuropathy, whereas others restrictive cardiomyopathy, or the clinical presentation may be mixed [5,6]. Most of the mutations are rare and only run in a couple of families, but others occur endemically like ATTRVal30Met in Portugal or ATTRGlu89Gln in Bulgaria [7].

The THAOS registry of ATTRv provides epidemiological information from the US, South America, some Asian countries, and Western Europe, but information about ATTRv in Eastern and Central Europe, apart from the endemic region in Bulgaria, was scarce until the recent publication of Austrian data [8].

Specific targeted pharmacological treatment for ATTRv is already available beyond liver and heart transplantation [9,10,11,12]. Therefore, identifying patients with ATTRv is crucial to improve their quality of life and life expectancy. Understanding the connection between genotype and phenotype may help define the patients who benefit the most from certain treatments.

The analysis of more than 5000 systemic amyloid patients from the UK from the past decades shows that the proportion of AL amyloidosis remained unchanged at about 67%, but the number of AA amyloidosis cases significantly dropped, and the number of new cases of ATTRwt greatly increased. The proportion of ATTRv was 6.6% in this large systemic amyloid population [1]. No epidemiological data are available from Hungary about the incidence or prevalence of the different types (AL, ATTRwt, ATTRv, and AA) of amyloidosis. However, authors have experienced similar changes in disease prevalence in Hungary as well. Until now, only a few case studies of Hungarian ATTRv patients have been published. The aim of this retrospective study was the analysis of the prevalence, regional distribution, genotype, and phenotype of Hungarian patients with ATTRv, and comparison of the results with available Central European data.

## 2. Materials and Methods

### 2.1. Patients and Design of Analysis

With the cooperation of the Hungarian regional and university cardiology, neurology, and rare disease centers, we retrospectively identified all patients with the genetically confirmed diagnosis of ATTRv. Only patients with known pathological mutations were included, based on the published mutation database [13]. We collected all available epidemiologic and clinical data and analyzed them retrospectively. Serum levels of natriuretic peptides were collected from the time of the diagnosis, where available.

Patient epidemiologic data included family history, sex, date of birth and death—if deceased, residence, and the type of ATTRv mutation. Mutations were named according to the latest recommendations of the International Society of Amyloidosis (ISA) Nomenclature Committee [4].

Clinical data included the age at the time of clinical diagnosis, time (months) from the first disease-specific symptom, as observed by the patient, to the clinical diagnosis of ATTRv, and the initial clinical presentation (cardiac, neurologic, mixed, or other phenotype). The time of diagnosis was defined as the time when all diagnostic criteria were fulfilled according to the current guidelines [14,15,16].

Where information was available, the primary cause of death was recorded as one of the following categories: cardiac (arrhythmia, heart failure progression), neurologic, other, or unknown. Heart transplantation (HTX) was performed in one case, but in the statistical analysis, it was considered as “cardiac death due to HF progression,” just as HTX is usually considered as “death” in clinical HF trials as endpoint. It was also noted whether the patients were symptomatic or asymptomatic at the time of the genetic test result of *TTR* mutation. The latter are patients identified as part of family screening. These patients were only included in the epidemiologic part of the analysis.

We also performed a search in publications: PubMed ((Hungary) OR (Hungarian)) AND ((*TTR*) OR (transthyretin)) to further identify Hungarian patients with ATTRv.

### 2.2. Cardiac Involvement of ATTRv

The cardiac involvement of ATTRv patients was established by retrospective analysis according to the suggested algorithm of the recent consensus recommendations [14,15]:

- histological diagnosis of cardiac amyloidosis—endomyocardial biopsy: endomyocardial biopsy positive for cardiac amyloidosis with Congo red staining with apple-green birefringence under polarized light; typing by immunohistochemistry and/or mass spectrometry at specialized centers
- histological diagnosis of cardiac amyloidosis—extracardiac biopsy: ATTR cardiac amyloidosis is diagnosed when extracardiac biopsy confirms ATTR amyloidosis AND typical cardiac imaging features are present [15])
- clinical diagnosis of ATTR cardiac amyloidosis—99mTc-PYP, DPD, HMDP: ATTR cardiac amyloidosis is diagnosed when cardiac scintigraphy (99mTc-PYP, DPD, HMDP) shows positive (grade 2 or 3) myocardial uptake of radiotracer AND clonal plasma cell dyscrasias are excluded by serum free light chains (FLCs) measurements and serum and urine immunofixation, AND typical cardiac imaging features are present [15])

In recent years, cardiac amyloidosis was diagnosed primarily by a non-biopsy method. The NYHA (New York Heart Association) functional class was used to grade heart failure severity at the time of clinical diagnosis. The leading cardiac symptoms in ATTRv are the symptoms of HF: dyspnea, fatigue, exercise intolerance, refractory volume overload, hypotension, and signs of inadequate perfusion.

### 2.3. Neurologic Involvement of ATTRv

Neurologic involvement was established retrospectively based on the patients’ neurologic evaluation including the electrophysiological assessment of peripheral nerves if clinical symptoms suggested polyneuropathy. CTS was not considered as a primary neurologic manifestation, because it is not a sign of polyneuropathy. For grading the severity of polyneuropathy at the time of clinical diagnosis, the modified polyneuropathy disability (PND) score was employed, a score specifically created for FAP patients [17]:

- I: sensory disturbances in the lower extremities, but the walking ability of the patient is not affected
- II: patient has some difficulty in walking, but does not require a walking aid
- III: patient requires a walking aid—A: one stick/crutch required; B: two sticks/crutches required
- IV: patient needs a wheelchair for mobility or is confined to bed

The leading neurologic symptoms in ATTRv are the symptoms of polyneuropathy: symmetric distal sensory loss, burning sensations, and weakness.

### 2.4. Genetic Testing of the TTR Gene

In one family with ATTRAsp18Gly, published 25 years earlier, the genetic testing was performed in New York, NY, USA, and the method was described in the original article [17]. In all other cases, the genetic testing was performed in Hungary, either at the Department of Internal Medicine, University of Szeged, or at the Department of Internal Medicine and Haematology, Semmelweis University, Budapest. At both universities, all four coding exons and exon–intron boundaries of the gene encoding transthyretin (TTR; OMIM# 176300) were amplified by polymerase chain reaction using genomic DNA purified from peripheral blood samples as templates and were directly sequenced with the BigDye Terminator v3.1 Cycle Sequencing Kit (Life Technologies Corporation, Carlsbad, USA) on an ABI Prism 310 Genetic Analyzer (Applied Biosystems, Waltham, Massachusetts, USA). The samples of unaffected family members served as normal controls. Sequencing electropherograms were analyzed by the Sequencing Analyzer software version v5.4. Variants in the TTR gene were annotated based on the reference sequence LRG_416. However, amino acid changes were reported according to the latest recommendations of the International Society of Amyloidosis (ISA) Nomenclature Committee, which use the mature protein in numbering of amino acid residues, i.e., without leader sequences. In Budapest and Szeged, in all cases, patients and family members/caregivers gave prior written informed consent to the molecular genetic analysis. Primer sequences and PCR conditions are available upon request.

### 2.5. Literature Search

The literature search was performed in PubMed using the following terms: “((Hungary) OR (Hungarian)) AND ((*TTR*) OR (transthyretin))”.

## 3. Results

### 3.1. Epidemiology of ATTRv in Hungary

**Figure 1:**
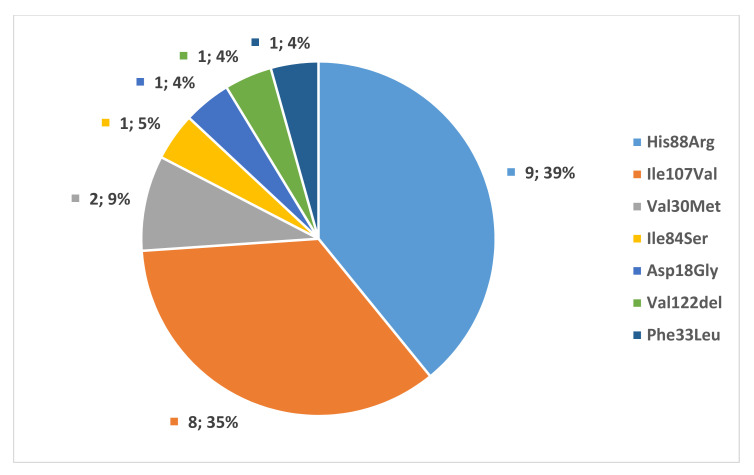
Genetic spectrum of *TTR* mutations in Hungarian families, expressed in number and percentage of the affected families (*n* = 23).

**Figure 2:**
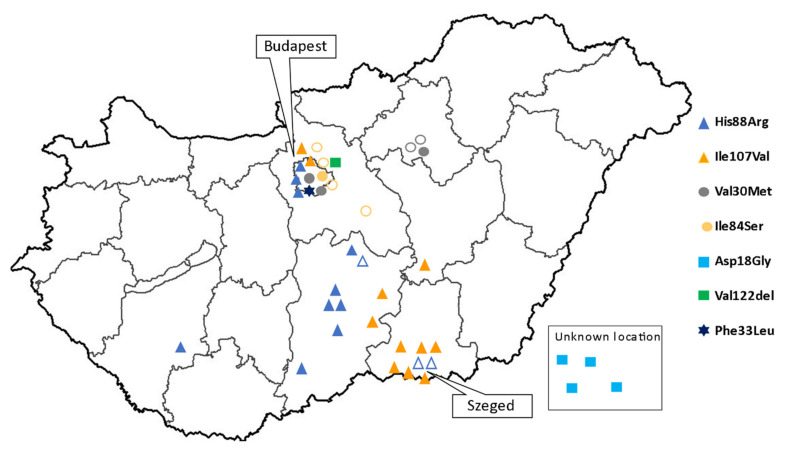
A map of Hungary with geographic distribution of different genetic subtypes of ATTRv patients (*n* = 40). Full symbols represent symptomatic individuals, while empty symbols represent asymptomatic individuals. The four patients with unknown locations are the patients found during literature search.

Twenty-two families with 36 individuals of known pathogenic *TTR* mutations were identified. Twenty-four patients were symptomatic, and 12 were asymptomatic at the time of the diagnosis. The four patients with only CTS were not considered symptomatic in the analysis, as CTS is not a manifestation of polyneuropathy. Seven different pathogenic mutations were found: ATTRHis88Arg (9 families, 13 individuals), ATTRIle107Val (8 families, 11 individuals), ATTRVal30Met (2 families, 5 individuals), ATTRVal122del (1 family, 1 individual), ATTRPhe33Leu (1 family, 1 individual), ATTRIle84Ser (1 family, 5 individuals)—summarized in Figure 1. The geographical distribution is shown in Figure 2. All patients were born in Hungary and were native Hungarians. Although all major Hungarian cardiology, neurology, and rare disease centers were contacted, the majority of patients originated from two regions as shown in Figure 1.

The search in PubMed revealed only one additional Hungarian family with four affected individuals. The clinical manifestations in this family with ATTRAsp18Gly mutation were published in several articles over 20 years ago [18,19,20,21,22]. Briefly, central nervous system involvement was the leading symptom in this family, without clinical signs of polyneuropathy and HF. Because detailed clinical data are not available today from this family, they were only included in the epidemiologic analysis. It should be noted that some of the cases in our retrospective analysis were already published elsewhere. Two of these case reports were published in international journals, which can be searched online [23,24].

### 3.2. Clinical Characteristics of ATTRv Patients in Hungary

**Figure 3:**
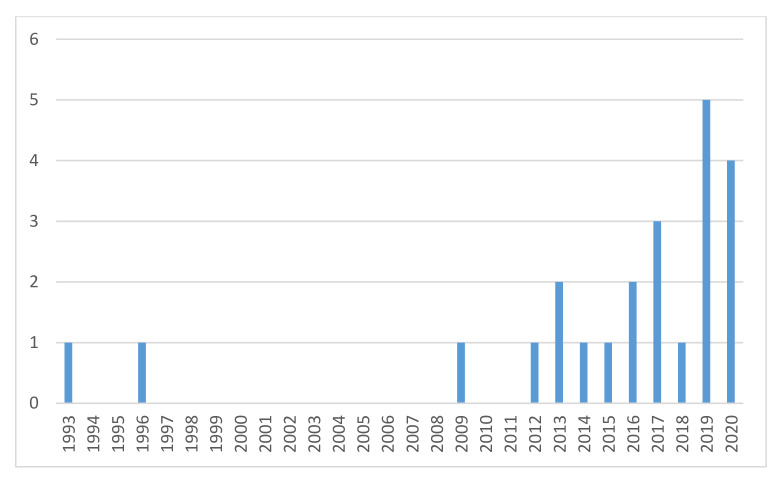
Number of families identified with ATTRv in recent years (*n* = 23).

The clinical findings of the 24 symptomatic patients are summarized in Table 1. The family of four affected members with ATTRAsp18Gly, identified with literature search, was excluded from this analysis, because clinical information was unavailable. Twelve individuals without clinical signs of HF or polyneuropathy were considered asymptomatic carriers. Four of them had CTS, and eight had no clinical manifestation at all, and all were diagnosed during family screening and are now followed regularly. Their median age was 29 years at the time of genetic testing. The median age of symptomatic patients at the time of clinical diagnosis was 65 years. The median time to clinical diagnosis from the first clinical symptoms was 54.8 months. All 24 patients had cardiac and 20 had neurologic involvement at the time of the clinical diagnosis. Seventeen patients had CTS in their medical history. In eight cases, the cardiac symptoms were more severe than the symptoms of polyneuropathy (only three had cardiac symptoms). In 11 cases, the neurologic symptoms were dominant over cardiac symptoms, but all had some degree of heart failure as well. In three cases, the symptoms of HF and polyneuropathy were equally severe. In two cases, data were insufficient. At the time of diagnosis, three, eight, eight, and two patients were in NYHA I, II, III, and IV functional stages, respectively. In four cases, data were missing. NT-proBNP values were available from the time of the diagnosis in 15 cases, with a median value of 3511 pg/mL. Patients with neurological involvement had late-onset length-dependent sensorimotor axonal polyneuropathy with PND score of I, II, III, and IV in eight, eight, three, and zero patients, respectively. Three patients had no polyneuropathy, data were unavailable in one case, and data were insufficient to determine the PND stage in another case. At the time of the analysis, 12 patients had already died and 1 patient had undergone HTX. Causes of death and the HTX: arrhythmia (1 case), progression of heart failure (6 cases), other (3 cases), or unknown (3 cases). No patient died due to the progression of polyneuropathy. Ten patients were on targeted pharmacological therapy at the time of the analysis or previously. Clinical data are summarized in Table 1. The timeline of family identification with ATTRv is shown in Figure 3.

In case of the most common ATTRHis88Arg mutation, the median age of the patients at the time of the clinical diagnosis was 62 years. The median time to clinical diagnosis was 43.8 months, which means that symptoms (excluding CTS) started at the median of 58.35 years. Median NYHA stage was III; median PND stage was II. Regarding the second most common ATTRIle107Val mutation, the median age at diagnosis was 73 years, and the median time to diagnosis was 66.7 months, indicating that symptoms started at the median age of 67.44 years. Median NYHA stage was II; median PND stage was I. The phenotypes of these two common Hungarian mutations are compared and summarized in Table 2.

## 4. Discussion

This is the first report of the prevalence, epidemiology, and geno- and phenotypes of Hungarian ATTRv patients. Apart from Bulgaria, where the ATTRGlu89Gln mutation is endemic, these data in the Eastern and Central European region were available only for Austria, from a recently published study [8]. To the best of our knowledge, all known families and patients with ATTRv in Hungary were captured in this retrospective analysis.

The major finding of this study is the demonstration of ATTRHis88Arg and ATTRIle107Val mutations as the most common pathogenic *TTR* mutations in Hungary. Limited knowledge is available concerning these mutations. ATTRIle107Val was considered as a mixed-phenotype mutation in a review in 2013 [6], but ATTRHis88Arg was recognized only in 2019 as a pathogenic mutation in ATTRv guidelines [25]. ATTRIle107Val was reported in many countries: for example, in Japan [26,27], France [28,29,30], Brazil [31], and Germany [32], although in a recent review of ATTRv, only 11 individuals were identified with ATTRIle107Val in Western Europe [33]. Data showed that the phenotype associated with ATTRIle107Val ATTRv is mixed: cardiac and neurologic features are both present, although the heart is predominantly involved [6,25]. This correlates with our findings in the Hungarian population, where all eight symptomatic ATTRIle107Val patients had a mixed phenotype, although in five cases polyneuropathy was the leading symptom as shown in Table 2.

ATTRHis88Arg was identified first in Sweden recently, where they found that all seven individuals originated from the same region and were related [34]. In their cases, cardiomyopathy and HF were the leading symptoms, but neurologic involvement was also observed. Among the 10 Hungarian symptomatic patients with ATTRHis88Arg ATTRv, 4 patients had heart failure, and 4 had polyneuropathy as the first symptom. Two had both HF and polyneuropathy at onset. However, at the time of diagnosis all had already some degree of heart failure. Among those seven patients who died during the follow-up, the cause of death was cardiac in five cases. This mutation was also found in one family recently in Asia [35]. ATTRHis88Arg mutation was reported also as the most common variant in a recent Austrian publication affecting six families [8]. This similarity in the frequency of ATTRHis88Arg in Austria and in Hungary may be explained by the common historic background of the Austro-Hungarian Empire. ATTRHis88Arg is a rare mutation and was probably introduced in the Austro-Hungarian population by a common ancestor. This theory of “founder effect” should be investigated in the future. A pooled analysis of the Austro-Hungarian patients would further refine our knowledge on the clinical characteristics of ATTRHis88Arg ATTRv, such as expected penetrance, age of onset, rate of progression in asymptomatic individuals as it is shown in other ATTRv-s [36,37].

One of the goals of this retrospective analysis was to determine the phenotype–genotype correlations for the most common Hungarian mutations. Although the absolute numbers of our cases may seem to be low regarding the two most common mutations, these numbers are high, when comparing them to case numbers from other publications. It is interesting that the age of ATTRHis88Arg patients at diagnosis was almost 10 years younger than in case of ATTRIle107Val patients, and they were also in a more advanced stage of the disease, with more severe HF and higher PND scores (Table 2). This suggests that ATTRHis88Arg is a more aggressive form of ATTRv than ATTRIle107Val. This observation may have therapeutic consequences in the future.

At the time of our analysis, the prevalence of ATTRv in Hungary was 2.35 per 1 million, which is lower than the 5.2 cases per million in Europe [8], not considering the endemic regions. Although the rising awareness for ATTR resulted in a significant increase in the number of new cases in recent years, as shown in Figure 3, it is possible that the disease is still underdiagnosed in Hungary, accounting for this difference. The number of newly diagnosed cases will probably increase further as the “red flags” for amyloidosis are actively spread among cardiologists and neurologists [25,38]. The geographical distribution of ATTRv in Hungary is shown in Figure 2. The inhomogeneity in the territorial distribution does not necessarily reflect real regional differences, but is more likely related to the higher probability of diagnosis in the two university hospitals of Budapest and Szeged, specialized in the care of patients with amyloidosis and genetic cardiomyopathies.

The evaluation of the mortality, prognosis, disease progression, and therapy is out of the scope of this article, as these parameters are affected by many factors. Most of the families were diagnosed in recent years (Figure 2) with only a short follow-up. Furthermore, our patient population was inhomogeneous regarding disease specific therapy, with 10 patients receiving tafamidis. One patient is on a daily dose of 61 mg (approved for ATTR cardiomyopathy); the others take 20 mg (approved for stage I ATTRv polyneuropathy). One patient participates in a placebo-controlled clinical trial, receiving either placebo or vutrisiran. Thirteen (54%) symptomatic patients died during the follow-up period ranging from 1 to 195 months, but median survival time cannot be calculated due to the short follow-up period of most surviving patients.

Our study has limitations. In some cases, due to the retrospective nature of the study, relevant laboratory and clinical data were missing. Four members of one family with ATTRvAsp18Gly who were identified by literature search were only included in the epidemiological analysis. Furthermore, taking into account the lower prevalence of Hungarian ATTRv cases in comparison to the non-endemic regions of Western Europe, undiagnosed cases may still be present in Hungary despite the efforts in increasing awareness of the disease.

## 5. Conclusions

This is the first analysis of epidemiology, prevalence, and geno- and phenotypes of the Hungarian ATTRv patients. ATTRHis88Arg was found to be the most common mutation in Hungary, similar to the neighboring Austria, which is extremely rare in other parts of the world. ATTRHis88Arg and ATTRIle107Val mutations encompass three quarters of the affected families, and essentially all patients have a mixed phenotypic manifestation of the disease. ATTRHis88Arg seems to be more aggressive form of ATTRv than ATTRIle107Val. The lower rate of prevalence in our analysis compared to data coming from Western Europe suggests that the disease is still underdiagnosed in Hungary.

## Figures and Tables

**Table 1:** Clinical characteristics of patients with symptomatic ATTRv (*n* = 24).

Sex		Male	Female
		18	6
Age at clinical diagnosis (median)		65 years	
Time to clinical diagnosis (median)		54.8 months	
Initial presentation (at the time of onset of first symptom)	cardiac	33.33% (*n* = 8)	
	neurologic	45.83% (*n* = 11)	
	mixed	12.5% (*n* = 3)	
	N/A	8.33% (*n* = 2)	
Stage of heart failure according to NYHA functional class at time of clinical diagnosis	I	12.5% (*n* = 3)	
	II	33.33% (*n* = 8)	
	III	33.33% (*n* = 7)	
	IV	8.33% (*n* = 2)	
	N/A	12.5% (*n* = 3)	
Median value of NT-proBNP at time of clinical diagnosis		3511 pg/mL (*n* = 15)	
PND at the time of clinical diagnosis	no polyneuropathy	12.5% (*n* = 3)	
	I	33.33% (*n* = 8)	
	II	33.33% (*n* = 8)	
	III	12.5% (*n* = 3)	
	IV	0% (*n* = 0)	
	N/A	8.33% (*n* = 2)	
CTS in medical history	positive history	75% (*n* = 18)	
	negative history	20.83% (*n* = 5)	
	N/A	4.16% (*n* = 1)	
Total number of patients receiving targeted pharmacological therapy		41.6% (*n* = 10) *	

NYHA: New York Heart Association—functional classification of heart failure; PND: modified polyneuropathy disability score; CTS: carpal tunnel syndrome; N/A: not available. * 1 additional patient participated in a placebo-controlled clinical trial.

**Table 2:** Genotype–phenotype correlation in symptomatic Hungarian ATTRv patients with the two common mutations (ATTRHis88Arg and ATTRIle107Val).

		ATTRIle107Val *n* = 8	ATTRHis88Arg *n* = 10
Number of patients with	heart failure as the leading symptom	2	4 *
	equally severe cardiac and neurologic symptoms	1	2
	polyneuropathy as the leading symptom	5	4
Median age at clinical diagnosis		73 years	62 years
Median time from first symptoms to diagnosis		66.7 months (*n* = 8)	43.8 months
Median age at first symptoms		67.44 years	58.35 years
Median NYHA stage		III	II
median PND stage		II	I

NYHA: New York Heart Association—functional classification of heart failure; PND: modified polyneuropathy disability score. * All patients with ATTRIle107Val and ATTRHis88Arg had mixed phenotypes, except one patient with only cardiac symptoms.

## Data Availability

The datasets used and/or analyzed during the current study are available from the corresponding author on reasonable request.
